# Modeling of drying kiwi slices and its sensory evaluation

**DOI:** 10.1002/fsn3.414

**Published:** 2016-08-13

**Authors:** Abbas Mahjoorian, Mohsen Mokhtarian, Nasrin Fayyaz, Fatemeh Rahmati, Shabnam Sayyadi, Peiman Ariaii

**Affiliations:** ^1^ Department of Food Science and TechnologyAyatollah Amoli BranchAzad Islamic UniversityAmolIran; ^2^ Young Researchers and Elite ClubSabzevar BranchIslamic Azad UniversitySabzevarIran; ^3^ Department of Food Science and TechnologyFaculty of AgricultureFerdowsi University of MashhadMashhadIran

**Keywords:** Artificial neural network, hot‐air drying, sensory evaluation

## Abstract

In this study, monolayer drying of kiwi slices was simulated by a laboratory‐scale hot‐air dryer. The drying process was carried out at three different temperatures of 50, 60, and 70°C. After the end of drying process, initially, the experimental drying data were fitted to the 11 well‐known drying models. The results indicated that Two‐term model gave better performance compared with other models to monitor the moisture ratio (with average *R* ^2^ value equal .998). Also, this study used artificial neural network (ANN) in order to feasibly predict dried kiwi slices moisture ratio (*y*), based on the time and temperature drying inputs (*x*_1_, *x*_2_). In order to do this research, two main activation functions called *logsig* and *tanh*, widely used in engineering calculations, were applied. The results revealed that, *logsig* activation function base on 13 neurons in first and second hidden layers were selected as the best configuration to predict the moisture ratio. This network was able to predict moisture ratio with *R* ^2^ value .997. Furthermore, kiwi slice favorite is evaluated by sensory evaluation. In this test, sense qualities as color, aroma, flavor, appearance, and chew ability (tissue brittleness) are considered.

## 1 Introduction

Kiwi plant (*Actinidia deliciosa*) is native to the Yangtze River valley of northern China and Zhejiang Province on the coast of eastern China (http://www.crfg.org/pubs/ff/kiwifruit.html). Annual production rate of kiwi has reached ~70,617 MT in 2012 to which Iran has contributed 32,000 MT (FAO, 2009). This crop is consumed in various ways as fresh nourishing, dried thin layers, and so on. Many studies have been conducted in field of drying of various fruits and vegetables. For instance, Guiné, Pinho, and Barroca (2011) worked on pumpkin behavior during drying. In this research, the experimental data were fitted to different models for moisture ratio and it was concluded that the best models were Page and modified Page. As well, Simal, Femenia, Garau, and Rossello (2005) studied kinetic drying of kiwi fruit with three mathematical models such as exponential, page, and diffusion models. The results showed that page model provided the best simulation of kiwi fruit drying curves. Orikasa, Wu, Shiina, and Tagawa (2008) studied characteristics of kiwi drying at four different temperatures. The highest hardness of surface and destruction of l‐ascorbic acid were evaluated during convective hot‐air drying. Doymaz and Ismail (2011) studied modeling of sweet cherry drying characteristics. The result showed that Page model was presented as the best model to describe cherry drying characteristics. Nowadays, neural network has an important role as a powerful tool in predicting process parameters. Artificial neural networks (ANNs) are modern calculating methods to predict output response from complex systems. The main idea of these network species was adapted from biological neural system functions in data processing for extending knowledge and information (15). A group of Iranian researchers applied multilayer perceptron (MLP) and radial basis function (RBF) networks for prediction drying kinetic parameters of pumpkin during hot‐air drying (Mokhtarian & Koushki, 2012). The other groups of researchers studied ANNs and Genetic algorithms to predict free fat content, lactose crystallization, and particle size mean during whole milk powder with spray drying (Koc, Heinemann, & Ziegler, 2007). Goni, Oddone, Segura, Mascheroni, and Salvadori (2008) utilized ANNs to predict food freezing time and thawing. Poonnoy, Tansakul, and Chinnan (2006) predicted temperature and moisture content of tomato slices during drying by microwave‐vacuum dryer. Momenzadeh, Zomorodian, and Mowla (2011) predicted drying time of corn shell during drying by concurrent microwave‐fluidized bed dryer by ANNs. Madadlou et al. (2009) predicted casein micelles size by combinative approach ANNs‐RSM. Mateo, Gadea, Mateo, and Jiménez (2011) used ANNs for predicting rate of accumulating Deoxynivalenol in infected barely seeds to Fusarium culmorum fungi.Fernandes et al. (2011) determined anthociyanin concentration in whole grape skin by ANNs and image processing. Lertworasirikul and Saetan (2010) used artificial neural network modeling to predict mass transfer parameters of kaffir lime peel (i.e., water loss and solid gain). ANNs were utilized to predict moisture ratio of dried potato slices by different drying methods in this project.

The aim of this research was studying kinetic drying of kiwi slices and investigating organoleptic characteristics of products during drying. Also feasibility prediction of kiwi moisture ratio with ANN was studied and the obtained results were compared with experimental models.

## 2 Material and Methods

### 2.1 Raw material preparation

In this research, fresh kiwi samples (Monty variety) were purchased from local market. Then samples were sorted as to color, diameter, and weight (wg: 135 g and diameter was 4–5 cm). In order to reduce respiration intensity and physiological and chemical changes, all samples were preserved in refrigerator at temperature of 5–6°C. At the beginning of each experiment, kiwi was washed with fresh water to remove the kiwi fines adhered to the fruit surface and cut into ring with a diameter of 5 cm and thickness of 3 ± 0.1 mm. The initial moisture content was determined by drying in hot‐air convective oven (Memmert, model UNE 400 PA, Scheabach, Germany) at 105°C for 48 hr (AOAC, 1990).

### 2.2 Drying equipment

A laboratory convective hot‐air dryer (Model Of‐02G; JEIO TECH, Seoul, Korea) was applied in the experiments. The experiments were carried out at three temperatures 50, 60, and 70°C. The relative humidity of the ambient air (∼30°C) was around 62–65%. The dryer was adjusted to a congenial temperature and became constant for 1.5 hr before the outset of a conduct test. When the fan motor switch was turned on, air passing an air inlet would distribute inside the chamber and then passed through an air outlet. The weight loss was monitored by means of a digital balance (Jewelry, AND, model FX‐CT SERIES, FX‐300 CT; Tokyo, Japan) through a sampling interval of 30 min and an accuracy of ±0.001 g undergoing drying. The final weight and moisture content of the kiwi slices were measured at the end of each air‐drying experiment. Drying was finished when the moisture content of the samples was about 0.15 ± 0.5 (kg water/kg dry matter).

### 2.3 Mathematical modeling of drying process

Monolayer drying models of experimental data of kiwi slices were expressed in the form of moisture ratio of samples during monolayer drying and it was displayed as (Eqn. (1)):

(1) $$\text{MR}=\frac{M-M_{e}}{M_{0}-M_{e}}$$

In these equations, MR, *M*,* M_0_*,* M_e_*, and *M*_*t*+d*t*_ are the moisture ratio, moisture content at any time, initial moisture content, equilibrium moisture content, moisture content at *t,* and moisture content at *t* + d*t* (kg water/kg dry matter), respectively, and *t* is drying time (min). Drying runs were done in triplicate. Mathematical models were simulated by software Sigma Plot Ver.11 (statistical software package, SigmaPlot, version 11, London, UK). The drying curves obtained were fitted with three different moisture ratio models (Table 1) (Togrül & Pehlivan, 2002).

**Table 1:** Drying kinetic models

Equation	Name
Newton	MR = exp(−*kt*)
Page	MR = exp(−*kt* ^*n*^)
Henderson and Pabis	MR = *a* exp(−*kt*)
Logarithmic	MR = *a* exp(−*kt*) + *c*
Two‐term	MR = *a* exp(−*k*_0_ *t* + *b* exp(−*k*_1_ *t*)
Two‐term exponential	MR = *a* exp(−*kt*) + (1−*a*) exp(−*kat*)
Wang and Singh	MR = 1 + *at* + *bt* ^2^
Diffusion approximation	MR = *a* exp(−*kt*) + (1−*a*) exp(−*kbt*)
Verma et al.	MR = *a* exp(−*kt*) + (1−*a*) exp(−*gt*)
Diffusion of Fick's	MR = *a* exp(−*c*(*t/L* ^2^))
Modified page II	MR = *a* exp(−*c(t/L* ^2^)^*n*^)

The coefficient of determination (*R* ^2^) was one of the main criteria for selecting the best equation. In addition to the coefficient of determination, the goodness of fit was determined by various statistical indicators such as reduced chi square (*χ* ^2^), mean relative deviation modulus *P* (%), and root mean square error (*RMSE*). For quality fit, *R* ^2^ value should be higher and *χ* ^2^, *P* (%), and *RMSE* values should be lower (Goyal & Bhargava, 2008). The above parameters can be calculated as follows:

(2) $$\chi^{2}=\frac{\sum_{i=1}^{N}{\left({\text{MR}}_{e,i}-{\text{MR}}_{p,i}\right)}^{2}}{N-z}$$

(3) $$P\left(\%\right)=\frac{100}{N}\sum_{i=1}^{N}\left|{\text{MR}}_{p,i}-{\text{MR}}_{e,i}\right|$$

(4) $$RMSE={\left[\frac{1}{N}\sum_{i=1}^{N}\left({\text{MR}}_{p,i}-{\text{MR}}_{e,i}\right)\right]}^{\frac{1}{2}}$$

where MR_*e,i*_ is experimental moisture ratio, MR_*p,i*_ is predicted moisture ratio, *N* is number of experimental data, and *z* is number of model parameters.

### 2.4 Computation of effective diffusion coefficient and activation energy

The experimental drying data were done for the determination of diffusivity coefficients by Fick's second diffusion equation. The analytical solution of Fick's second law is unsteady‐state diffusion in an infinite slab by the drying process seen in the Equation (5):

(5) $$\text{MR}=\frac{M-M_{e}}{M_{o}-M_{e}}=\frac{8}{\pi^{2}}\exp\left[\frac{-\pi^{2}D_{{}^{{\mathrm{eff}}^{t}}}}{4L^{2}}\right]$$

where MR is moisture ratio (dimensionless), *D*_eff_ is the effective diffusion coefficient (m^2^/s), and *L* is the half‐thickness of sample (*m*). Therefore, effective diffusion coefficient (*D*_eff_) was obtained by plotting ln MR versus time (min). From Equation (5), a plot of ln MR versus time displayed a straight line with a slope of (α), in which (Eqn. (6)):

(6) $$\alpha =\frac{\pi^{2}D_{\mathrm{eff}}}{4L^{2}}$$

### 2.5 Sensory evaluation test

Sensory evaluation test was carried out with a group containing 10 educated panelists. All evaluations were done through Single Stimulus method and five scores hedonic tests. The prepared questionnaires with four questions were asked to everyone and for every question there were five options. The proposed questions were color acceptance, odor and flavor, appearance shape, and chewiness (texture crispiness). Every one marked as own self taste one of choice such as very good, good, fair, poor, and very poor. Finally, scoring was done for every choice separately (very good = 5, good = 4, fair = 3, poor = 2, and very poor = 1). In this research, multiple Duncan's test in 0.01 level was analyzed with SAS software 9.1 version (Lawless & Heymann, 2010).

### 2.6 Artificial neural network (ANN)

An artificial neural network composed of simple processing elements called neurons that are connected to each other by weights. The neuron is grouped into distinct layers and interconnected according to a given architecture (Mousavi & Javan, 2009). A multilayer perception (MLP) networks is one of the most popular and successful neural network architectures, suited to wide range of engineering application involved drying.

Mathematically:

(7) $$y_{j}=\sum_{i=1}^{n}f\left(w_{ij}x_{i}\right)+b_{j}$$

where *y*_*j*_ is the net input of each neuron in the hidden and output layers, *x*_*i*_ is input, *n* is number of inputs to the neuron, *w*_*ij*_ is the weight of the connection between neuron *I,* and neuron *j* and *b*_*j*_ is the bias associated with *j*th neuron (Mohebbi, Shahidi, Fathi, Ehtiati, & Noshad, 2011). Each neuron consists of a transfer function expressing its internal activation level. Output from a neuron is determined by transforming its input using a suitable transform function.

Generally, the transfer functions are sigmoidal, guassian, hyperbolic tangent, hyperbolic secant, and linear functions. Sigmoidal (*logsig*) and hyperbolic tangent (*tanh*) functions were used to establish nonlinear relationship in engineering applications (Picton, 2000):

(8) $$logsig\left(z\right)=\frac{1}{1+\exp(-z)}\;\left(0,+1\right)$$

(9) $$tahn\left(z\right)=\frac{e^{z}-e^{-z}}{e^{z}+e^{-z}}\;\left(-1,+1\right)$$

**Figure 1:**
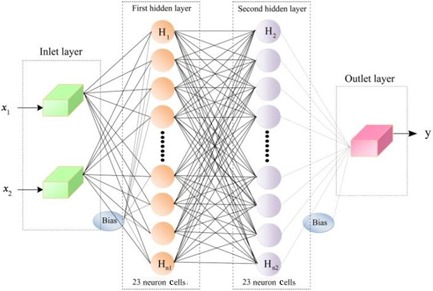
Schematic structure of perceptron neural network, where *x*_1_ is drying temperature, *x*_2_ is drying time, and *y* is moisture ratio (MR)

As can be seen, Figure 1 shows schematic structure of perceptron neural network. In this network, the input layer consists of two neurons (air temperature (*x*_1_) and drying time (*x*_2_)) and the output layer contains one neuron (moisture ratio (*y*)).

The back propagation algorithm was used in training of ANN model. This algorithm uses the supervised training technique where the network weights and biases are initialized randomly at the beginning of the training phase (Singh & Pandey, 2011). In order to optimize ANN, different factors including hidden layer number, neuron number per hidden layer, type of activation function in hidden and output layers, learning rate, and momentum coefficients must be evaluated. In this work, number of 1–2 hidden layers with 5–15 neurons per hidden layer, learning rate = 0.4, momentum coefficient = 0.9, and activation functions of sigmoid logarithms (Eqn. (8)) and hyperbolic tangent (Eqn. (9)) in each hidden and output layer were used in order to find the best topology.

In order to network modeling, data were randomly divided into two groups, 70% used for training and reminder 30% for testing of network. Data modeling accomplished by using SPSS statistical software version 19 (2011). For determination of the best network arrangement two criteria of determination were used, coefficient (*R* ^2^) and mean relative error (*MRE*), respectively:

(10) $$R^{2}=1-\left[\frac{{\sum_{i=1}^{N}\left(P_{\mathrm{ANN},i}-P_{\exp\;,i}\right)}^{2}}{{\sum_{i=1}^{N}\left({\bar{P}}_{\mathrm{ANN},i}-P_{\mathrm{ANN},i}\right)}^{2}}\right]$$

(11) $$MRE=\left(\frac{1}{N}\sum_{i=1}^{N}\frac{\left|\left(P_{\mathrm{ANN},i}-P_{\exp\;,i}\right)\right|}{P_{\exp\;,i}}\right)\times 100$$

where *P*_ANN_ is predicted ANN output parameter, *P*_exp_ is experimental data, and *N* is number of observations.

## 3 Results and Discussion

### 3.1 Mathematical modeling of drying kinetic

**Figure 2:**
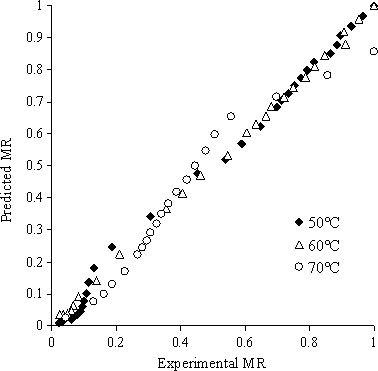
The results comparison of predicted and experimental moisture ratio for best dynamic model of kiwi monolayer drying

Eleven dynamic models of kiwi monolayer drying were processed at temperature of 50, 60, and 70°C. Statistical parameters of tests were *R* ^2^, χ^2^, *RMSE*, and PE (%). Statistical analysis amounts were presented for each parameter of Tables 2, 3, and 4 in brief. *R* ^2^ value for processed models was higher than 0.8307 in all cases. Changes amplitude of *R* ^2^ was between .8307 and .9996, χ^2^ was 0.0000157 and 0.0178, and variety range amount of *RMSE* was 0.00384 and 0.1269, respectively, and also variety range amount of PE (%) was 2.85 and 83.8, respectively. Statistical analysis results showed that Two‐term model had the highest *R* ^2^ and lowest χ^2^, *RMSE*, and PE (%). Therefore, increasing *R* ^2^ and lowering of χ^2^, *RMSE* was the most important cause in selecting the best processed model. Comparison experimental and predicted moisture ratio data have been represented for the best processed model in temperature range 50–70°C and have been represented in Figure 2. Predicted data for moisture ratio in experimental data as direct line on diagram show that the obtained Two‐term dynamic model can better describe characteristics of kiwi drying. Similar results were observed by Doymaz and Ismail (2011).

**Table 2:** The statistic result of kiwi monolayer drying at 50°C

Model	PE (%)	*RMSE*	χ^2^	*R* ^2^
Newton	25.49	0.02986	0.000921	.9964
Page	22.09	0.02801	0.000838	.9968
Henderson and Pabis	24.88	0.02947	0.000928	.9965
Logarithmic	15.29	0.0190	0.0004	.9985
Two‐term	14.33	0.01894	0.000412	.9986
Two‐term exponential	19.01	0.02546	0.000692	.9974
Wang and Singh	83.80	0.10883	0.012661	.951
Diffusion approximation	24.88	0.02947	0.000962	.9965
Modified page II	22.09	0.02801	0.000868	.9968
Verma et al.	14.50	0.01908	0.000403	.9985
Diffusion of Fick's	14.50	0.01908	0.000403	.9985

**Table 3:** The statistic result of kiwi monolayer drying at 60°C

Model	PE (%)	*RMSE*	χ^2^	*R* ^2^
Newton	12.36	0.0143	0.000215	.9991
Page	12.05	0.0143	0.000223	.9991
Henderson and Pabis	12.49	0.0143	0.000223	.9991
Logarithmic	4.38	0.010	0.000114	.9995
Two‐term	4.85	0.0098	0.000115	.9996
Two‐term exponential	9.89	0.0132	0.000189	.9992
Wang and Singh	65.68	0.0837	0.007625	.9674
Diffusion approximation	12.49	0.0143	0.000234	.9991
Modified page II	12.05	0.0143	0.000233	.9991
Verma et al.	5.28	0.0103	0.000121	.9995
Diffusion of Fick's	5.28	0.0103	0.000121	.9995

**Table 4:** The statistic result of kiwi monolayer drying at 70°C

Model	PE (%)	*RMSE*	χ^2^	*R* ^2^
Newton	24.3	0.0767	0.006181	.9417
Page	7.81	0.0258	0.000737	.9936
Henderson and Pabis	16.17	0.0593	0.003893	.9655
Logarithmic	19.84	0.0445	0.002312	.9807
Two‐term	5.09	0.0154	0.000293	.9977
Two‐term exponential	15.46	0.0506	0.002834	.975
Wang and Singh	49.64	0.1268	0.017798	.8307
Diffusion approximation	16.17	0.0593	0.004110	.9655
Modified page II	7.81	0.0258	0.000778	.9936
Verma et al.	5.15	0.0160	0.0003	.9975
Diffusion of Fick's	5.15	0.0160	0.0003	.9975

**Figure 3:**
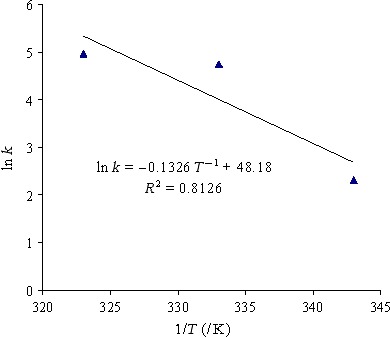
Arrhenius‐type relationship between log kinetic parameter of Two‐term model versus inverse temperature

In addition, parameters of various applied models are presented in Table 5. Kinetic constant (*k*) increased as drying air temperature increased, thus could be predicted by Arrhenius relationship (Eqn. (12)). By taking logarithm from both side of that equation and plot ln *k* versus *T* ^−1^, first‐order regression fitted data with a high determination coefficient (.9251) as shown in Figure 3.

**Table 5:** Parameters of applied models at different temperatures

Model names	Temperature (℃)	*k* (min^−1^)	*n*	*a*	*b*	*c*	*h*	*l*	*g*
Newton	50	0.00611	–	–	–	–	–	–	–
	60	0.00830	–	–	–	–	–	–	–
	70	0.02230	–	–	–	–	–	–	–
Page	50	0.00831	0.9341	–	–	–	–	–	–
	60	0.00856	0.9932	–	–	–	–	–	–
	70	0.09110	0.6313	–	–	–	–	–	–
Henderson and Pabis	50	0.00596	–	0.9886	–	–	–	–	–
	60	0.00834	–	1.0019	–	–	–	–	–
	70	0.0180	–	0.8569	–	–	–	–	–
Logarithmic	50	0.00684	–	0.9585	–	0.0462	–	–	–
	60	0.00888	–	0.9838	–	0.0256	–	–	–
	70	0.0304	–	0.7845	–	0.1468	–	–	–
Two‐term	50	0.00696	–	0.9461	0.0597	–	0.00034	–	–
	60	0.00870	–	0.9975	0.0106	–	−0.0017	–	–
	70	0.0988	–	0.4413	0.5771	–	0.0107	–	–
Two‐term exponential	50	0.00941	–	0.4492	–	–	–	–	–
	60	0.0102	–	0.5954	–	–	–	–	–
	70	0.0857	–	0.203	–	–	–	–	–
Wang and Singh	50	–	–	−0.0033	2.6 × 10^−6^	–	–	–	–
	60	–	–	−0.0053	6.7 × 10^−6^	–	–	–	–
	70	–	–	−0.0148	5.5 × 10^−5^	–	–	–	–
Diffusion approximation	50	0.00022	–	0.0535	30.60	–	–	–	–
	60	0.00850	–	0.9934	−0.294	–	–	–	–
	70	0.094	–	0.4263	0.1129	–	–	–	–
Modified page II	50	–	0.9341	–	–	0.013	–	−1.2725	–
	60	–	0.9932	–	–	0.0064	–	0.8643	–
	70	–	0.6313	–	–	0.0687	–	0.8002	–
Verma et al.	50	0.00682	–	0.9465	–	–	–	–	0.00022
	60	−0.00249	–	0.0066	–	–	–	–	0.00850
	70	0.0106	–	0.5737	–	–	–	–	0.0940
Diffusion of Fick's	50	–	–	0.9887	–	0.0043	–	−0.8585	–
	60	–	–	1.002	–	48.831	–	−76.51	–
	70	–	–	0.8569	–	0.0665	–	1.9243	–

(12) $$k=k_{o}\exp\left(\frac{E_{a}}{RT}\right)$$

**Figure 4:**
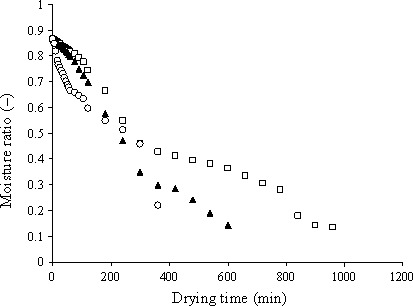
Curve variation in kiwi moisture ratio versus drying time at different temperature during drying (drying temperature □ = 50°C, ▲ = 60°C, O = 70°C)

The results showed that drying air temperature had a significant effect on the drying time. This status is clearly observed in drying curve (Fig. 4). As clear from diagram, the essential time for kiwi drying from primary moisture content 86.6% (wet basis) to final moisture content 5% (wet basis) was 960, 600, and 360 min for temperature of 50, 60, and 70, respectively. Similar results were observed by other researchers for different vegetables (Doymaz & Ismail, 2011).

Effective moisture diffusion of kiwi was calculated in 50, 60, and 70°C, 4.55 × 10^−11^, 9.12 × 10^−11^, 21.27 × 10^−11^, respectively. As observed, effective moisture diffusion of kiwi in 70°C was higher than 60°C in similar conditions. This context is described through mass transfer from capillary pores of food. Similar results have been represented in different vegetables in Table 6.

**Table 6:** Comparison of effective moisture diffusion values of kiwi fruit and other crops

Crops	*D*_eff_ (m^2^/s)	Temperature range (°C)	Reference
Carrot	0.77–9.33 × 10^−9^	50–70	Doymaz (2004a)
Apricot	6.76–12.6 × 10^−10^	55	Doymaz (2004b)
Sweet cherry	1.54–5.68 × 10^−10^	60–75	Doymaz and Ismail (2011)
Kiwi	4.55–21.27 × 10^−11^	50–70	This study

### 3.2 Sensory analysis of kiwi slices during drying

**Figure 5:**
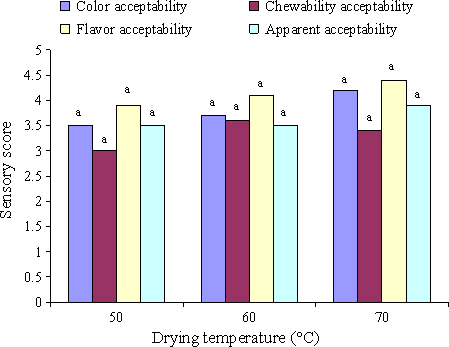
The result of kiwi slices sensory evaluation during drying

The results of sensory evaluation of kiwi slices have been represented in different temperature ranges in Figure 5. The results demonstrated that among color acceptance, odor, taste, and texture crispiness (chewiness), no significant difference was observed (*p* < .01) and the dried sample had the highest sensory score at 70°C. Its cause was due to browning reactions that led to the sample color change and this problem has been realized by panelists. The results showed that there was no significant difference between odor and taste and the dried sample had the highest score at 70°C. Appearance acceptance of the dried slices showed that there was no significant difference among all samples at 50, 60, and 70°C and the dried sample had the highest sensory score at 70°C. Related to texture crispiness acceptance (chewiness), the results showed that there was no significant difference among the studied samples in probability level 99%, although the dried sample in 60°C had the highest statistical score.

### 3.3 Artificial neural network modeling

In this research, a combination of different layers and neurons with different activation functions were used for modeling perceptron neural networks. Neural network with one and two hidden layers, 3–15 neurons were selected randomly and network power was estimated to predict kiwi moisture ratio.

**Figure 6:**
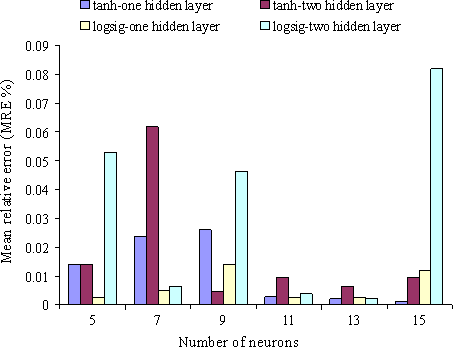
Variation in relative error amount against neurons number to predict moisture ratio

The results of optimization of perceptron neural network with logarithm sigmoid activation function and hyperbolic tan with obtained rearrangements have been represented in different cases in Figure 6. Figure 6 shows the variation in relative error value versus number of neuron to predict moisture ratio. Investigating the obtained results especially multilayer perceptron neural network with *logsig* activation function showed that neural network with 2‐13‐13‐1 rearrangement, i.e., network with two inputs, 13 neurons in the first and second hidden layer, and one output had the best result in predicting the moisture ratio (regression coefficient value in this case was .997). On the other hand, the results of perceptron neural network with hyperbolic tan activation function showed that neural network with 2‐15‐1 had the best results in predicting the moisture ratio as this network could estimate moisture ratio during monolayer drying process of kiwi with regression coefficient =.994 (relative error amount was calculated as 0.001092 in this case).

**Figure 7:**
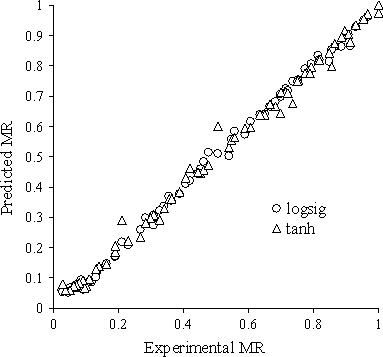
The predicted and experimental values of perceptron neural network with *logsig* and *tanh* activation function to predict moisture ratio of tentative sample

Model sensitivity diagram of predicted parameters by MLP network with *logsig* and *tanh* activation functions against experimental parameters for the best topology are shown in Figure 7. Result comparison of different activation function of ANN shows that *logsig* activation function with 13 neurons in first and second hidden layer due to higher *R* ^2^ value (.997) was the best model to predict kiwi moisture ratio.

## 4 Conclusion

In this study, temperature effect was investigated on kiwi drying characteristic. Increasing drying temperature caused a reduction in time and raise of velocity and effective diffusion coefficient. After model statistical analysis, the results showed that Two‐term model is the best model for kiwi drying monolayer due to maximum *R* ^2^, minimum χ^2^, and *RMSE*, among those processed dynamic models. Moreover, product sensory characteristics were evaluated in this research by panelists. The results revealed that among color acceptance, odor, taste, and texture crispiness (chewiness) were not observed any significant differences in probability level =.99. Multilayer perceptron neural network with different functions was used to estimation feasibility of moisture ratio with neural network. The results showed that neural network with sigmoid log activation function contain 13 neurons in the first and second hidden layers that could predict moisture ratio amount with regression coefficient =.997.

## References

[fsn3414-bib-0001] AOAC . (1990). Official method of analysis, 15th ed., vol. 2. Arlington: Association of Official Analytical Chemitists Inc.

[fsn3414-bib-0002] Doymaz, I. (2004a). Convective air drying characteristics of thin layer carrots. Journal of Food Engineering, 61, 359–364.

[fsn3414-bib-0003] Doymaz, I. (2004b). Effect of pre‐treatments using potassium metabisulphide and alkaline ethyl‐oleate on the drying kinetics of apricots. Journal of Biosystems Engineering, 89, 281–287.

[fsn3414-bib-0004] Doymaz, I. , & Ismail, O. (2011). Drying characteristics of sweet cherry. Journal of Food and Bioproducts Processing, 89, 31–38.

[fsn3414-bib-0005] Fernandes, A. M. , Oliveira, P. , Moura, J. P. , Oliveira, A. A. , Falco, V. , Correia, M. J. , & Melo‐Pinto, P. (2011). Determination of anthocyanin concentration in whole grape skins using hyperspectral imaging and adaptive boosting neural networks. Journal of Food Engineering, 105, 216–226.

[fsn3414-bib-0006] Food and Agriculture Organization (FAO) . (2009). Fao Stat: Agriculture Data. Available from http://faostat.fao.org/site/567/DesktopDefault.aspx?PageID=567#ancor [accessed May, 2011].

[fsn3414-bib-0007] Goni, S. M. , Oddone, S. , Segura, J. A. , Mascheroni, R. H. , & Salvadori, V. O. (2008). Prediction of foods freezing and thawing times: Artificial neural networks and genetic algorithm approach. Journal of Food Engineering, 84, 164–178.

[fsn3414-bib-0008] Goyal, R. K. , Mujjeb, O. , & Bhargava, V. K. (2008). Mathematical modeling of thin layer dryingkinetics of apple in tunnel dryer. International Journal Food Engineering, 4, 1–16.

[fsn3414-bib-0009] Guiné, R. P. F. , Pinho, S. , & Barroca, M. J. (2011). Study of the convective drying of pumpkin (*Cucurbita maxima*). Journal of Food and Bioproducts Processing, 89, 422–428.

[fsn3414-bib-0010] Koc, A. B. , Heinemann, P. H. , & Ziegler, G. R. (2007). Optimization of whole milk powder processing variables with neural networks and genetic algorithms. Journal of Food and Bioproducts Processing, 85, 336–343.

[fsn3414-bib-0011] Lawless, H. T. , & Heymann, H. (2010). Sensory evaluation of food: Principles and practices. New York: Springer.

[fsn3414-bib-0012] Lertworasirikul, S. , & Saetan, S. (2010). Artificial neural network modeling of mass transfer during osmotic dehydration of kaffir lime peel. Journal of Food Engineering, 98, 214–223.

[fsn3414-bib-0013] Madadlou, A. , Emam‐Djomeh, Z. , Ebrahimzadeh Mousavi, M. , Ehsani, M. R. , Javanmard, M. , & Sheehan, D. (2009). Response surface optimization of an artificial neural network for predicting the size of re‐assembled casein micelles. Journal of Computers and Electronics in Agriculture, 68, 216–221.

[fsn3414-bib-0014] Mateo, F. , Gadea, R. , Mateo, E. M. , & Jiménez, M. (2011). Multilayer perceptron neural networks and radial‐basis function networks as tools to forecast accumulation of deoxynivalenol in barley seeds contaminated with Fusarium culmorum. Journal of Food Control, 22, 88–95.

[fsn3414-bib-0015] Mohebbi, M. , Shahidi, F. , Fathi, M. , Ehtiati, A. , & Noshad, M. (2011). Prediction of moisture content in pre‐osmosed and ultrasounded dried banana using genetic algorithm and neural network. Journal of Food and Bioproduct Processing, 89, 362–366.

[fsn3414-bib-0016] Mokhtarian, M. , & Koushki, F. (2012). Modeling of drying kinetic of pumpkin: Part II. Artificial neural approach. The 1st Middle‐East Drying Conference (MEDC2012). February 19–20, 2012, Mahshar, Iran.

[fsn3414-bib-0017] Momenzadeh, L. , Zomorodian, A. , & Mowla, D. (2011). Experimental and theoretical investigation of shelled corn drying in a microwave‐assisted fluidized bed dryer using artificial neural network. Journal of Food and Bioproducts Processing, 89, 15–21.

[fsn3414-bib-0018] Mousavi, M. , & Javan, S. (2009). Modeling and simulation of apple drying, using artificial neural network and neuro‐Taguchi's method. Journal of Agriculture Science and Technology, 11, 559–571.

[fsn3414-bib-0019] Orikasa, T. , Wu, L. , Shiina, T. , & Tagawa, A. (2008). Drying characteristics of kiwifruit during hot air drying. Journal of Food Engineering, 85, 303–308.

[fsn3414-bib-0020] Picton, P. (2000). Neural Networks. Palgrave: Macmillan Press.

[fsn3414-bib-0021] Poonnoy, P. , Tansakul, A. , & Chinnan, M. (2006). Artificial neural network modeling for temperature and moisture content prediction in tomato slices undergoing microwave‐vacuum drying. Journal of Food Engineering and Physical properties, 49, 185–191. 10.1111/j.1750-3841.2006.00220.x 17995884

[fsn3414-bib-0022] Simal, S. , Femenia, A. , Garau, M. C. , & Rossello, C. (2005). Use of exponential Page's and diffusional models to simulate the drying kinetics of kiwifruit. Journal of Food Engineering Progress, 66, 323–328.

[fsn3414-bib-0023] Singh, N. J. , & Pandey, R. K. (2011). Neural network approaches for prediction of drying kinetics during drying of sweet potato. Agricultural Engineering International: CIGR Journal, 13, 1–12.

[fsn3414-bib-0024] Togrül, I. T. , & Pehlivan, D. (2002). Mathematical modelling of solar drying of apricots in thin layers. Journal of Food Engineering, 55, 209–216.

